# Evaluation of the acute oral toxicity and antipsychotic activity of a dual inhibitor of PDE1B and PDE10A in rat model of schizophrenia

**DOI:** 10.1371/journal.pone.0278216

**Published:** 2022-12-01

**Authors:** Mayasah Al-Nema, Anand Gaurav, Ming Tatt Lee, Patrick Okechukwu, Piyarat Nimmanpipug, Vannajan Sanghiran Lee

**Affiliations:** 1 Faculty of Pharmaceutical Sciences, UCSI University, Kuala Lumpur, Malaysia; 2 Office of Postgraduate Studies, UCSI University, Kuala Lumpur, Malaysia; 3 Graduate Institute of Pharmacology, College of Medicine, National Taiwan University, Taipei, Taiwan; 4 Faculty of Applied Sciences, UCSI University, Kuala Lumpur, Malaysia; 5 Department of Chemistry, Faculty of Science, Chiang Mai University, Chiang Mai, Thailand; 6 Center of Excellence for Innovation in Analytical Science and Technology for Biodiversity-based Economic and Society (I-ANALY-S-T), Chiang Mai University, Chiang Mai, Thailand; 7 Department of Chemistry, Faculty of Science, University of Malaya, Kuala Lumpur, Malaysia; Univerity of Texas at Austin, UNITED STATES

## Abstract

Phosphodiesterase 1B (PDE1B) and PDE10A are dual-specificity PDEs that hydrolyse both cyclic adenosine monophosphate and cyclic guanosine monophosphate, and are highly expressed in the striatum. Several reports have suggested that PDE10A inhibitors may present a promising approach for the treatment of positive symptoms of schizophrenia, whereas PDE1B inhibitors may present a novel mechanism to modulate cognitive deficits. Previously, we have reported a novel dual inhibitor of PDE1B and PDE10A, compound 2 [(3-fluorophenyl)(2-methyl-2,3-dihydro-4H-benzo[b][1,4]oxazin-4-yl)methanone] which has shown inhibitory activity for human recombinant PDE1B and PDE10A *in vitro*. In the present study, the safety profile of compound 2 has been evaluated in rats in the acute oral toxicity study, as well as; the antipsychotic-like effects in the rat model of schizophrenia. Compound 2 was tolerated up to 1 g/kg when administered at a single oral dose. Additionally, compound 2 has strongly suppressed ketamine-induced hyperlocomotion, which presented a model for the positive symptoms of schizophrenia. It has also shown an ability to attenuate social isolation induced by chronic administration of ketamine and enhanced recognition memory of rats ​in the novel object recognition test. Altogether, our results suggest that compound 2 represents a promising therapy for the treatment of the three symptomatic domains of schizophrenia.

## Background

Schizophrenia is a chronic debilitating disorder affecting the cognition, perception, emotion and behaviour of patients. It is characterised by three symptomatic domains; positive symptoms, negative symptoms, and cognitive impairments. The severity of the disorder, signs and symptoms, and the effect of the disease on the patient’s quality of life may vary among individuals [1]. Although the aetiology of schizophrenia is not exactly clear, several studies have referred to dopamine as the main neurotransmitter involved in the pathophysiology of the disorder. Most of the dopamine neurons are located in the basal ganglia in the brain. In schizophrenia, the dopaminergic system of the basal ganglia shows several anomalies. Therefore, the basal ganglia are the primary target for currently available antipsychotic medications [2]. Dopamine system abnormality plays a critical role in psychosis, decreased cognition, abnormal reward function, and movement disorders, all of which are seen in patients with schizophrenia. The brain has three dopaminergic pathways, mesolimbic pathway, mesocortical pathway and nigrostriatal pathway. The dopamine neurons arising at the ventral tegmental area (VTA) project to the nucleus accumbens (NA) and prefrontal cortex via the mesolimbic and mesocortical pathways, respectively [3]. In schizophrenia, the positive symptoms are developed due to the hyperactivity of dopamine in the mesolimbic pathway [4–6]. While the negative symptoms and cognitive impairments developed due to the decreased level of dopamine in the mesocortical pathway [7]. In contrast, the dopamine neurons arising in substantia nigra (SN) project to the striatum (caudate and putamen) via the nigrostriatal pathway. The striatum is the main input nuclei in the basal ganglia. Various neurochemically distinct cell types are located in the striatum; however, the striatal medium spiny neurons (MSNs) are the most prevalent. The MSNs are two types that participate in two pathways in the basal ganglia, the direct pathway (express dopamine D_1_-receptors) or indirect pathways (express dopamine D_2_-receptors). The dopaminergic input from the SN exerts a dual effect on MSNs, where it inhibits the indirect pathway neurons and activates the direct pathway neurons. Thus, the decline in dopamine input from the SN to the striatum would stimulate the indirect pathway neurons and decrease the activation of the direct pathway neurons. As a consequence, increased inhibition from indirect MSNs to the globus pallidus externus, which then increases the inhibitory output neurons in the globus pallidus interna (GPi) and substantia nigra pars reticulate (SNr). In parallel, decreased activation of direct MSNs reduces the inhibition of the GPi and SNr, which increase the activation of inhibitory basal ganglia output. Therefore, the alterations in the basal ganglia function result in the development of extrapyramidal adverse effects [8, 9].

There are 65 antipsychotics utilised across the world, which are categorised as typical antipsychotics and atypical antipsychotics [10, 11]. All antipsychotics control the positive symptoms of schizophrenia by blocking the D_2_-receptors, but there is insufficient evidence to support the efficacy of these medications to improve the negative symptoms and cognitive deficits [12, 13]. Furthermore, typical antipsychotics are known to cause extrapyramidal side effects by excessive block of D_2_-receptors in the striatum. Whereas, atypical antipsychotics are associated with metabolic adverse effects like weight gain and diabetes, which affects the patient’s adherence to medications [14, 15]. Therefore, novel treatment with potent efficacy against the three symptomatic domains of schizophrenia and a better safety profile would be of considerable therapeutic value.

Inhibition of cyclic nucleotide phosphodiesterase (PDE) may provide a new therapeutic approach for the treatment of schizophrenia disorder [15]. PDEs are a group of enzymes encoded by 21 genes and subdivided into 11 families based on structural and functional properties [16, 17]. PDE enzymes are expressed in unique and overlapping patterns throughout the body and central nervous system (CNS) [18]. Among these PDEs, the PDE1B isoform is highly abundant in the striatum, prefrontal cortex and hippocampus. PDE1B is co-localised with dopamine D_1_-receptors; thus, it represents the major inactivation mechanism of D_1_-receptors in the striatum and prefrontal cortex [19]. The inhibition of PDE1B activity in the prefrontal cortex potentiates the D_1_-receptor signalling and mitigates the negative symptoms and cognitive impairments. While; the inhibition of PDE10A activity has generated much excitement as a potentially novel mechanism to treat the positive symptoms of schizophrenia. The PDE10A enzyme is highly expressed in the striatum in both direct and indirect pathway neurons (with a higher expression in the indirect pathway neurons) [20, 21]. The inhibition of PDE10A activity blocks the D_2_-receptor signalling and improves the positive symptoms. A number of selective PDE1B inhibitors and PDE10A inhibitors have been developed. However, none of these inhibitors have reached the market yet due to inefficiency or side effects in clinical trials [22]. According to previous studies, the PDE1B inhibitors have proven to be effective in treating the cognitive symptoms; however, these inhibitors don’t have effects on the positive symptoms [23]. In contrast, the PDE10A inhibitors are effective in treating the positive symptoms, but there is insufficient evidence to support the efficacy of these inhibitors on the negative and cognitive symptoms [24]. Therefore, a dual PDE1B and PDE10A inhibitor with a balanced activation of the direct and indirect pathway neurons might be well suited to treat the cognitive symptoms along with the positive and negative symptoms associated with deficient D_1_ dopaminergic signalling, and high D_2_ dopaminergic signalling exists in schizophrenia disorder without producing serious side effects. In which the PDE1B inhibitors potentiate the signalling of D_1_ receptors. Whereas; the PDE10A inhibitors block the signalling of D_2_ receptors.

We recently identified a novel dual inhibitor of PDE1B and PDE10A, compound 2 [(3-fluorophenyl)(2-methyl-2,3-dihydro-4H-benzo[b][1,4]oxazin-4-yl)methanone] and confirmed the inhibitory activity for PDE1B and PDE10A *in vitro*; the half-maximal inhibitory concentration was 0.85 μM and 1.34 μM for PDE1B and PDE10A, respectively [25]. In this study, we evaluated the safety profile of compound 2 and characterised the *in vivo* efficacy as a novel treatment of schizophrenia.

## Materials and methods

### Ethics statement

The care and use of the animals and the experimental protocols were conducted according to the Animal ethics guideline approved by Universiti Kebangsaan Malaysia Animal Use Ethics Committee (UKMAEC). All possible efforts were made to minimise the animals’ suffering and to reduce the number of animals used.

### Experimental animals

162 Sprague-Dawley (SD) male rats, weighing between 200–250 g and age 6–8 weeks old, were received from Sinar Scientific (Selangor, Malaysia). The rats were housed in three rats per cage (21”W, 15”D, 8”H) with wood shaving as bedding and maintained on 12h light/dark cycle in a ventilated room (temperature 25 ± 2°C) for one week prior to dosing to allow for acclimatisation to the laboratory conditions. The rats were fed with standard laboratory rat chow and free access to drinking water. Body weight was measured individually for each rat before starting the experiments to calculate the proper treatment dosage. The volume was adjusted based on the body weight of the rat using 10 ml/kg [26]. Prior to each experiment, rats were placed in the laboratory setting for at least 60 min for habituation.

### Drugs and chemicals

Compound 2 was procured from Alinda Chemical trade company limited, Moscow, Russia. Risperidone was procured from Sigma-Aldrich, St. Louis, USA. Ketamine was procured from Pet Arcade Sdn Bhd, Selangor, Malaysia. Dimethyl sulfoxide (DMSO) was purchased from Nacalai Tesque, Kyoto, Japan. Normal saline (0.9% sodium chloride) was purchased from Classic Chemicals Sdn Bhd, Selangor, Malaysia.

### Drug preparations

All the drugs were freshly prepared on the day of the experiment. Compound 2 was dissolved in 10% DMSO + 2% Tween 80 in saline, while risperidone was dissolved in 2% DMSO in saline. Ketamine (25 mg/kg) was prepared from stock solution (100 mg/ml). Vehicle (10% DMSO + 2% Tween 80 in saline) and drugs were dosed at 10 ml/kg body weight.

### Experimental design

**Fig 1:**
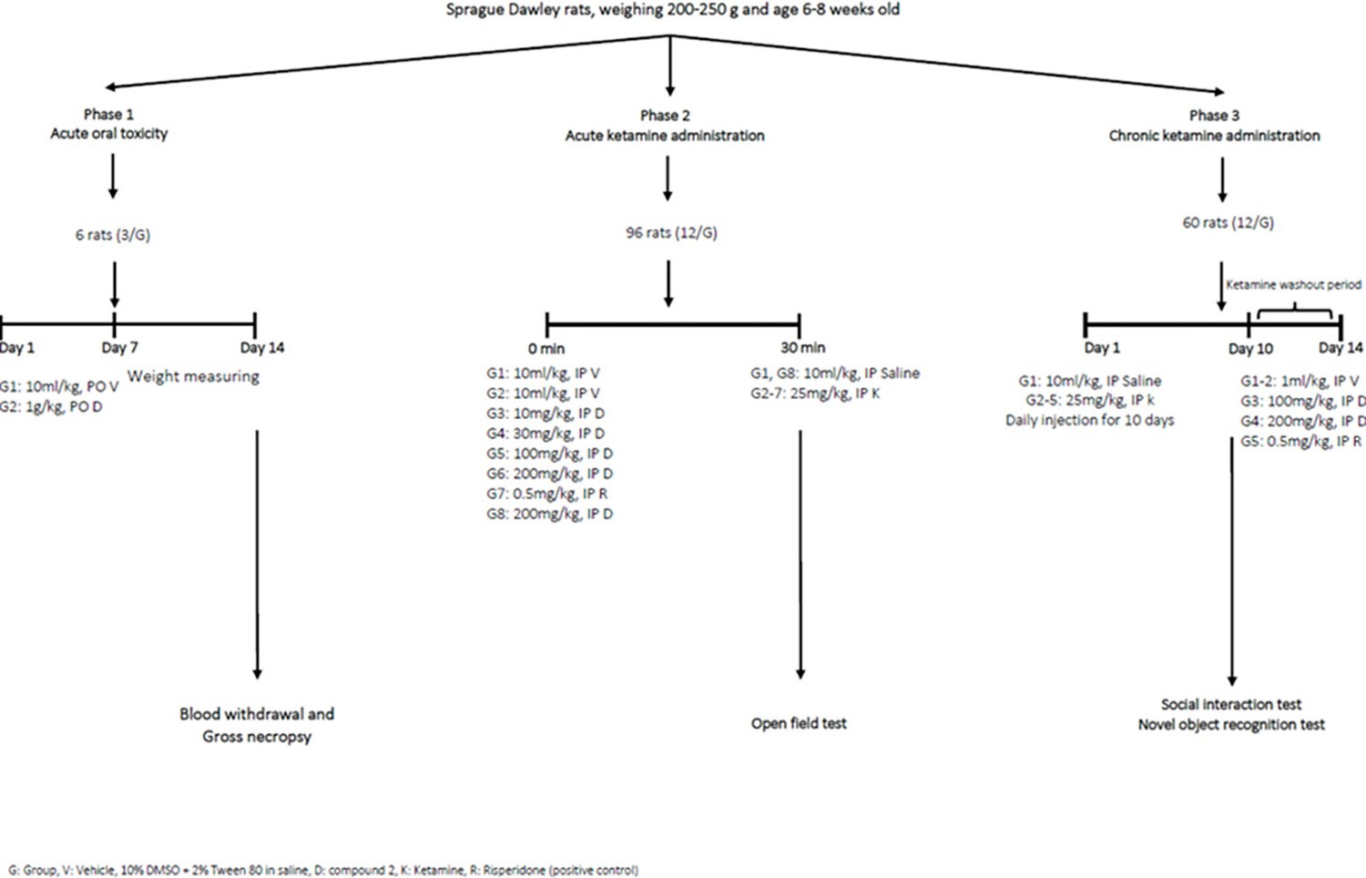
Flowchart of experimental work.

Acute oral toxicity study and behavioural tests (open field test, social interaction test, and novel object recognition test) were carried out in three phases (Fig 1).

Phase 1 (Acute Oral Toxicity): Six rats were divided into two groups, three rats per group. Group 1 received the vehicle, while group 2 received compound 2 (1 g/kg, p.o.).

Phase 2 (Acute Study): 96 rats were randomly divided into eight groups, 12 rats per group. Groups 1 and 2 received the vehicle. Groups 3–6 received compound 2 (10, 100 and 200 mg/kg, i.p.), while Group 7 received risperidone (0.5 mg/kg i.p.) and served as the positive control. Group 8 received compound 2 (200 mg/kg, i.p.). After 30 min, Groups 2–7 received ketamine (25 mg/kg, i.p.), whereas groups 1 and 8 received saline.

Phase 3 (Chronic Study): 60 rats were randomly divided into five groups, 12 rats per group. Group 1 received saline for ten days. Group 2–5 received ketamine (25 mg/kg, i.p.) for ten days to induce the negative and cognitive symptoms of schizophrenia. Ketamine was withdrawn after ten days of repeated treatment, and the rats were left undisturbed for three days for drug washout. On day 14th, groups 1–2 received vehicle, groups 3 and 4 received compound 2 (100 and 200 mg/kg, i.p.), and group 5 received risperidone (0.5 mg/kg i.p.).

### Phase one: Acute oral toxicity

The toxic effect of a single oral dose of compound 2 was evaluated according to the Organization for Economic Cooperation and Development (OECD) Guideline (Testing of Chemical No. 423) [27]. The rats were fasted overnight before treatment and until 4 h after treatment. Rats were observed during the first 60 min, hourly up to 6 h and once daily up to 14 days for any abnormal behaviour and sign of toxicity or mortality, in addition to changes in the skin, fur and eyes. The sign of toxicity includes abnormal behaviour like CNS depression (hypoactivity, relaxation and ataxia) and CNS stimulation (Hyperactivity, irritability, tremors and convulsion). The water intake, food consumption and defecation were also observed. The body weight was measured individually before and after treatment on day 1, 7 and 14, in which the change in body weight (%) was calculated according to the following equation [28]:

$$\frac{Body\;weight\;at\;the\;end\;of\;each\;week\;(g)‐initial\;body\;weight\;(g)}{Initial\;body\;weight\;(g)}x\;100$$

### Biochemical analysis

At the end of the acute oral toxicity experiment, the rats were anaesthetised on day 14^th^ by administration of intraperitoneal injection of anaesthetic solution (ketamine/xylazine, 100/10 mg/kg) [29]. A blood sample (5ml) was drawn from the orbital venous sinus using a heparinized capillary tube, followed by analysing the biochemical parameters, i.e. urea, creatinine, uric acid, total protein, albumin, alkaline phosphatase (ALP), alanine aminotransferase (ALT), aspartate aminotransferase (AST), sodium, potassium, and chloride. Rats were sacrificed by cervical dislocation immediately after blood collection [28].

### Macroscopic examination of vital organs

A gross necropsy was performed on both control and treatment groups rats following euthanasia by cervical dislocation on day 14^th^. The gross necropsy included careful examination of the external surfaces, orifices, thoracic cavity, abdominal cavity and their contents. The internal organs, i.e. heart, liver, kidney, spleen and lung, were excised through a midline incision in the rat’s abdomen. The organs were appropriately trimmed of any adherent tissue, blotted with clean tissue paper, then weighed and macroscopically examined for possible damage [28]. The relative organ weights were calculated according to the following formula [30]:

$$Relative\;organ\;weight\;(\%)=\frac{Organ\;weight\;(g)}{Final\;body\;weight\;of\;the\;rat\;(g)}x\;100$$

### Phase two: Acute study (Ketamine-induced hyperlocomotion)

#### Open field test

A graded doses analysis was carried out to identify the optimum dose of compound 2 that inhibits the ketamine-induced hyperlocomotion activity in rats. Following treatment with ketamine, the open field (OF) test was conducted, which is usually used to evaluate the animals’ locomotory and exploratory activity. The OF apparatus was purchased from (Print Mark Sdn Bhd, Kuala Lumpur, Malaysia). It is a squared arena made of black, matte, non-reflective Polyvinyl Chloride (PVC) foam with a closed base and an open-top (45cm x 45cm base, 40cm height). The rats were placed individually at the centre of the arena and allowed to explore the arena for 30 min. The total distance travelled by each rat in the OF arena was recorded during the 30 min. A Logitech C930e webcam was fixed to the ceiling directly facing the arena for recording. ANY-maze 6.3 video tracking software (anymaze.co.uk) was used for the analysis of rats’ behaviour. At the end of the test, the rat was removed from the arena and returned to its cage; and the arena was cleaned with 70% ethanol to remove the odour [31].

### Phase three: Chronic study (Ketamine-induced social isolation and cognitive impairments)

#### Social interaction test

The social interact test (SIT) was conducted to evaluate the effect of compound 2 on ketamine-induced social isolation. The amount of social interaction was measured as the amount of time spent on social exploration in the chamber containing the unfamiliarised rat in an open field arena. Initially, the tested rat was habituated to the testing apparatus, an arena made of black, matte, non-reflective PVC foam with a closed base and an open-top (45cm x 90cm base, 40cm height), by placing the rat in an empty arena for 15 min. The control rat (unfamiliarised rat) was habituated to the iron cage (11cm x 11cm base, x 23cm height) in advance of the experiment by placing the rat in a clean cage for 15 min. The SIT test was started 30 min after administration of the treatment. The test rat was placed in the centre of the arena that contains two wire cages at both ends, one with the control rat and the other one empty, and allowed to explore the arena for 15 mins. At the end of the 15 mins exploration time, the test rat was removed, and the arena was cleaned with 70% ethanol [32]. The rats were video recorded during the test and ANY-maze 6.3 was used to analyse the rats’ behaviour. The time spent in exploring the cages was measured, and the social preference was defined as follows: % time spent in the social chamber—% time spent in the opposite chamber [33]. The measuring parameters included: the time of the direct contact between the test rat and the control rat, in addition to the time spent by the test rat sniffing the cage with the control rat versus time spent sniffing the empty cage, which measures direct social interactions [34].

#### Novel object recognition test

To evaluate the effect of compound 2 on the recognition memory, which is known to be impaired in schizophrenia, the novel object recognition (NOR) test was conducted. The test consists of two sessions; sample trial and choice trial. The sample trial was started after the SIT, in which the rats were placed individually in the centre of the arena (same as SIT arena) containing two identical objects and allowed to explore the arena for 15 min. After 24 h, the rats were tested in the choice trial for recognition memory. The choice trial consists of a 15 min exploration of the field containing both a familiar object and a novel object [35]. The familiar object used here was a blue colour cylindrical glass container (29 cm diameter and 9 cm height), while the novel object was a red colour glass bottle (22 cm diameter and 17 cm height). The two objects were symmetrically fixed to the floor of the box with a distance of 14 cm from the walls. Objects and their placement into the open field were varied across rats to avoid positional biases. The objects and the floor of the arena were cleaned with 70% ethanol to control the odour cues after each testing session. The rats were video recorded during the sample and choice trials to ensure accuracy and reliability in the scoring of the behaviour. ANY-maze 6.3 was used for the analysis of rats’ behaviour. The exploration time was defined as an orientation toward the object with the nose of the rat within < 2 cm of the object. Licking, sniffing, or touching the object with forelimbs while sniffing was considered as exploration as well. Whereas leaning against the object to look upward and standing or sitting on the object were excluded. The object-discrimination index (DI) was defined as [36]:

$$DI=\frac{Novel\;exploration-Familiar\;exploration}{Total\;exploration}x\;100$$

* Memory score (DI × 100)

### Statistical analysis

Statistical analysis was performed using a statistical analysis program (SPSS, 16.0, International Business Machines, USA). Statistical significance between two groups was determined by independent samples t-test while significant effects across more than two groups were determined by One-way analysis of variance (ANOVA) followed by Tukey’s post hoc test to identify the differences among treatment means. The value of p < 0.05 was considered to be statistically significant. Data are presented as a mean ± S.E.M.

## Results

### Acute oral toxicity

**Fig 2:**
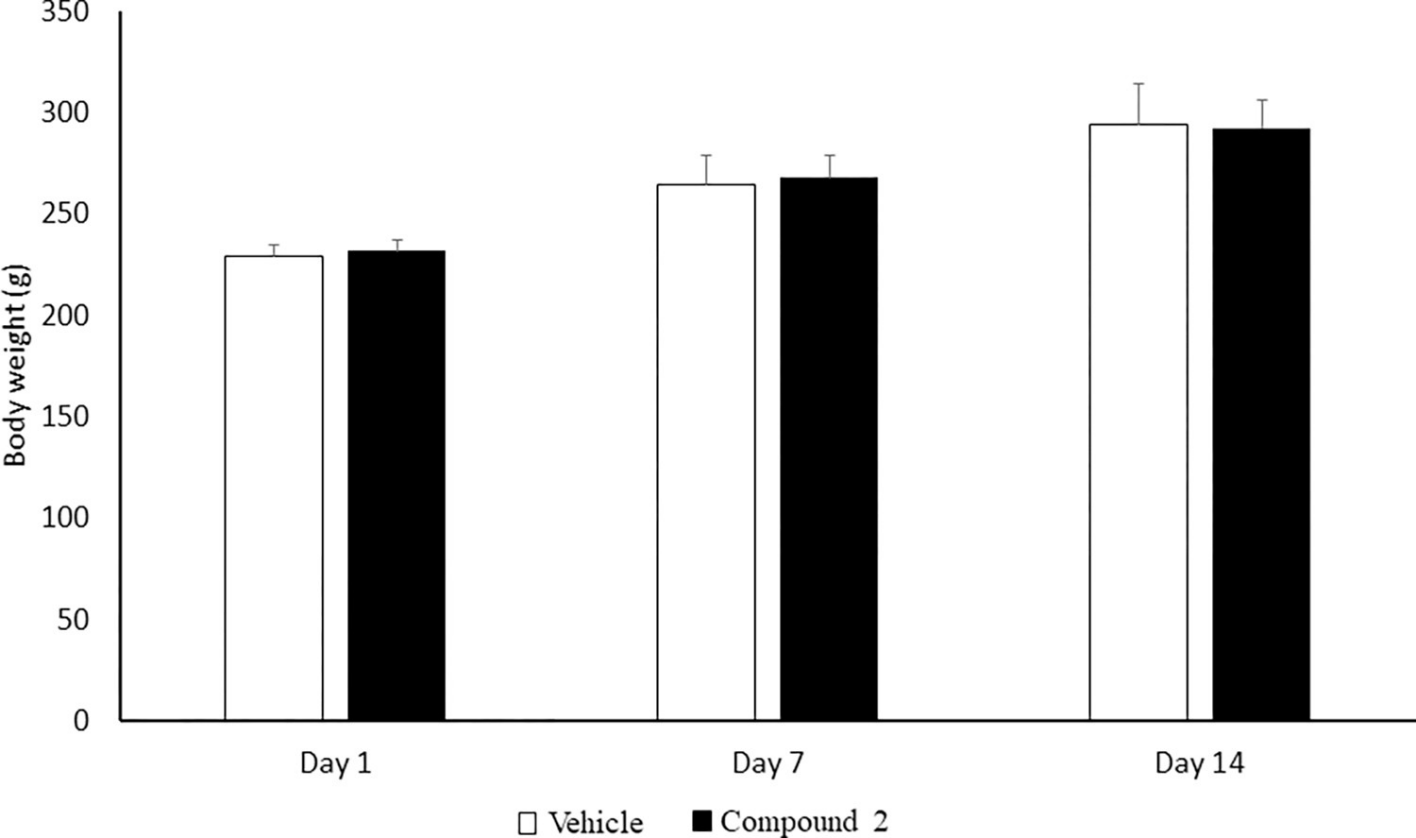
Mean body weight of vehicle and compound 2-treated rats. Body weight was measured at three intervals of the study period. Data are expressed as a mean ± standard error of mean (S.E.M) (n = 3).

The toxic effect of compound 2 at a dose of 1 g/kg on the appearance and the behaviour pattern of rats are presented in S1 Table. The results indicated no toxic symptoms or mortality were observed in any animal. All the rats presented the same appearance and behaviour and did not display any toxic symptoms, i.e. changes in breathing, water consumption, food intake, skin and hair loss and postural abnormalities throughout the 14-days observation period. Moreover, the gain in body weight was normal for all rats, as shown in Fig 2. There was a gradual increase in body weight of the control and compound 2-treated groups; in addition, the percentage changes in body weight of the compound 2-treated rats were not significantly different (p > 0.05) compared to the control rats (Table 1). In regard to necropsy examination, there was no statistically significant difference (p > 0.05) in the relative organ weight between the control and compound 2-treated group. Further, no treatment-related pathological changes of vital organs were observed in rats administrated compound 2 (Table 2).

**Table 1:** Body weight gain (%) of vehicle and compound 2-treated rats measured at three intervals of the study period.

Day	Vehicle	Compound 2 (1 g/kg)	*P* Value
**1**	228.97 g ± 6.01	231.71 g ± 5.83	
**7**	15.51% ± 3.10	15.61% ± 1.86	0.979
**14**	28.30% ± 5.27	26.14% ± 2.64	0.732

*Data are expressed as a mean ± S.E.M (n = 3) and analysed by independent samples t-test, p ˃ 0.05 for compound 2-treated group compared with the vehicle group.

**Table 2:** Relative organ weights (%) of vehicle and compound 2-treated rats in acute oral toxicity study.

Organ	Relative organ weights (%)		*P* value
	Vehicle	Compound 2 (1 g/kg)	
**Heart**	0.33 ± 0.01	0.30 ± 0.003	0.205
**Liver**	3.09 ± 0.8	2.89 ± 0.01	0.076
**Left kidney**	0.37 ± 0.01	0.34 ± 0.01	0.222
**Right kidney**	0.33 ± 0.01	0.31 ± 0.005	0.140
**Spleen**	0.29 ± 0.04	0.28 ± 0.01	0.835
**Lung**	0.81 ± 0.13	0.84 ± 0.03	0.875

*Data are expressed as a mean ± S.E.M (n = 3) and analysed by independent samples t-test, p ˃ 0.05 for compound 2-treated group compared with the vehicle group.

The data on biochemical parameters in control and compound 2-treated groups are presented in S2 Table. Generally, the administration of compound 2 did not produce treatment-related adverse effects on serum biochemistry parameters in rats. There was no statistically significant difference (p > 0.05) in the biochemical parameters between the control and treated groups. Thus, the non-toxic nature of compound 2 was evident by the absence of toxic symptoms and mortality of test animals at an oral dose of 1 g/kg.

### PDE1B/PDE10A inhibitor prevents ketamine-induced hyperlocomotion

**Fig 3:**
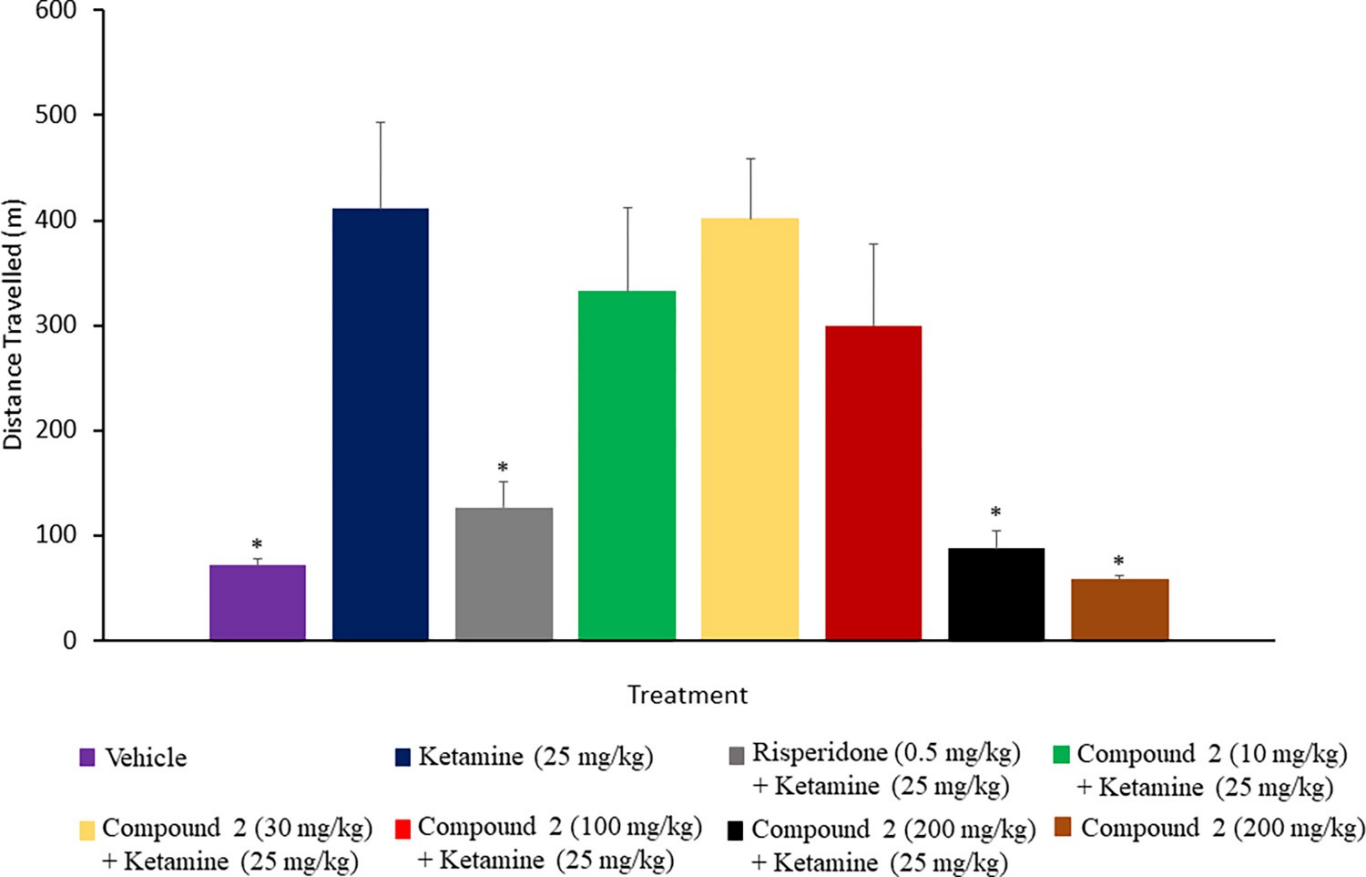
Effect of compound 2 on ketamine-induced hyperlocomotion in rats. Rats were pretreated with vehicle, risperidone or compound 2, 30 min before ketamine administration and then tested for locomotion activity. Rats treated with 200 mg/kg compound 2 showed a significant difference in locomotion activity compared to ketamine-treated rats. Data are expressed as a mean ± S.E.M (n = 8–12) and analysed by One-way ANOVA followed by Tukey’s post hoc test, *p < 0.05 compared to the schizophrenia control group.

**Fig 4:**
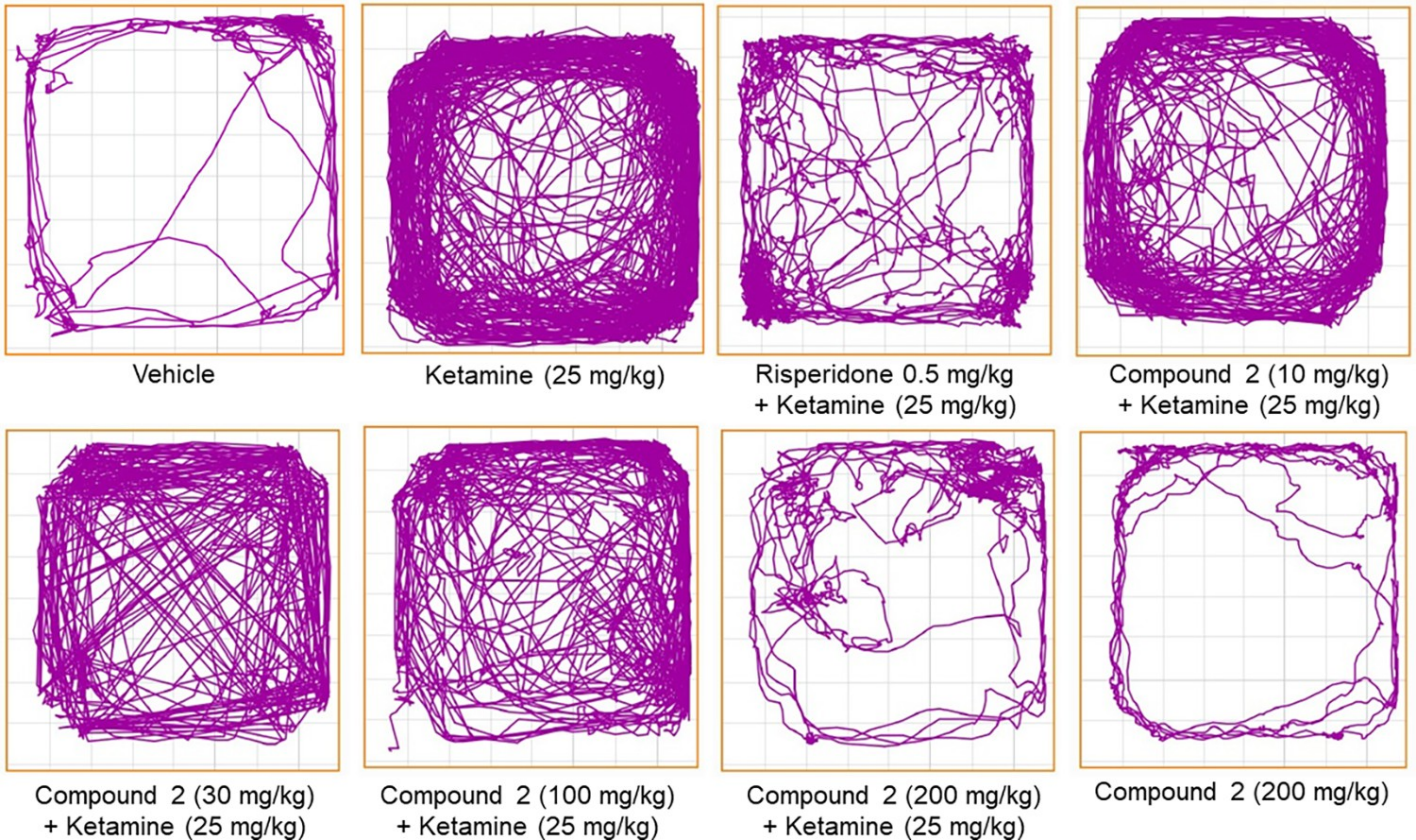
Representatives of track plots of locomotor activity of experimental groups of rats in open field test.

Ketamine-induced hyperlocomotion has commonly been used as an animal model for acute psychosis on the basis of the N-Methyl-D-Aspartate (NMDA) receptor hypofunction hypothesis of schizophrenia [33]. The effect of compound 2 and risperidone on motor activity in the open-field test is shown in Figs 3 and 4. One-way ANOVA has revealed a significant difference (p < 0.05) in the total distance travelled between the ketamine (25 mg/kg) group and the treatment groups. Accordingly, Tukey’s post hoc analysis showed that ketamine administration significantly (p < 0.05) increased the locomotion activity compared to the control group as indexed by the increase in the total distance travelled in the open field arena, where the mean distance value was 71.29 m and 411.16 m for control group and ketamine (25 mg/kg) group, respectively. The administration of compound 2 at doses of 10 and 100 mg/kg prior to ketamine slightly reduced the hyperlocomotion in which the compound 2 (10 mg/kg) group had a mean distance value of 332.57 m, and the compound 2 (100 mg/kg) group had a mean distance value of 299.99 m. In contrast, compound 2 at a dose of 30 mg/kg did not affect ketamine-induced hyperactivity, where the mean distance value was 401.49 m. However, pre-treatment with compound 2 (200 mg/kg) and risperidone (0.5 mg/kg) significantly (p < 0.05) inhibited the hyperactivity induced by ketamine when compared with the ketamine (25 mg/kg) group. The mean distance value was 87.16 m and 126.91 m for the compound 2 (200 mg/kg) group and risperidone group, respectively. Moreover, the effect of compound 2 alone on the basal locomotion was also assessed in the rats received the highest dose of compound 2 (200 mg/kg) before saline administration. In this group, the mean distance travelled of 58.24 m was close to the control group value and was significantly (p < 0.05) different from the ketamine (25 mg/kg) group.

### PDE1B/PDE10A inhibitor attenuates social isolation in ketamine-treated rats

**Fig 5:**
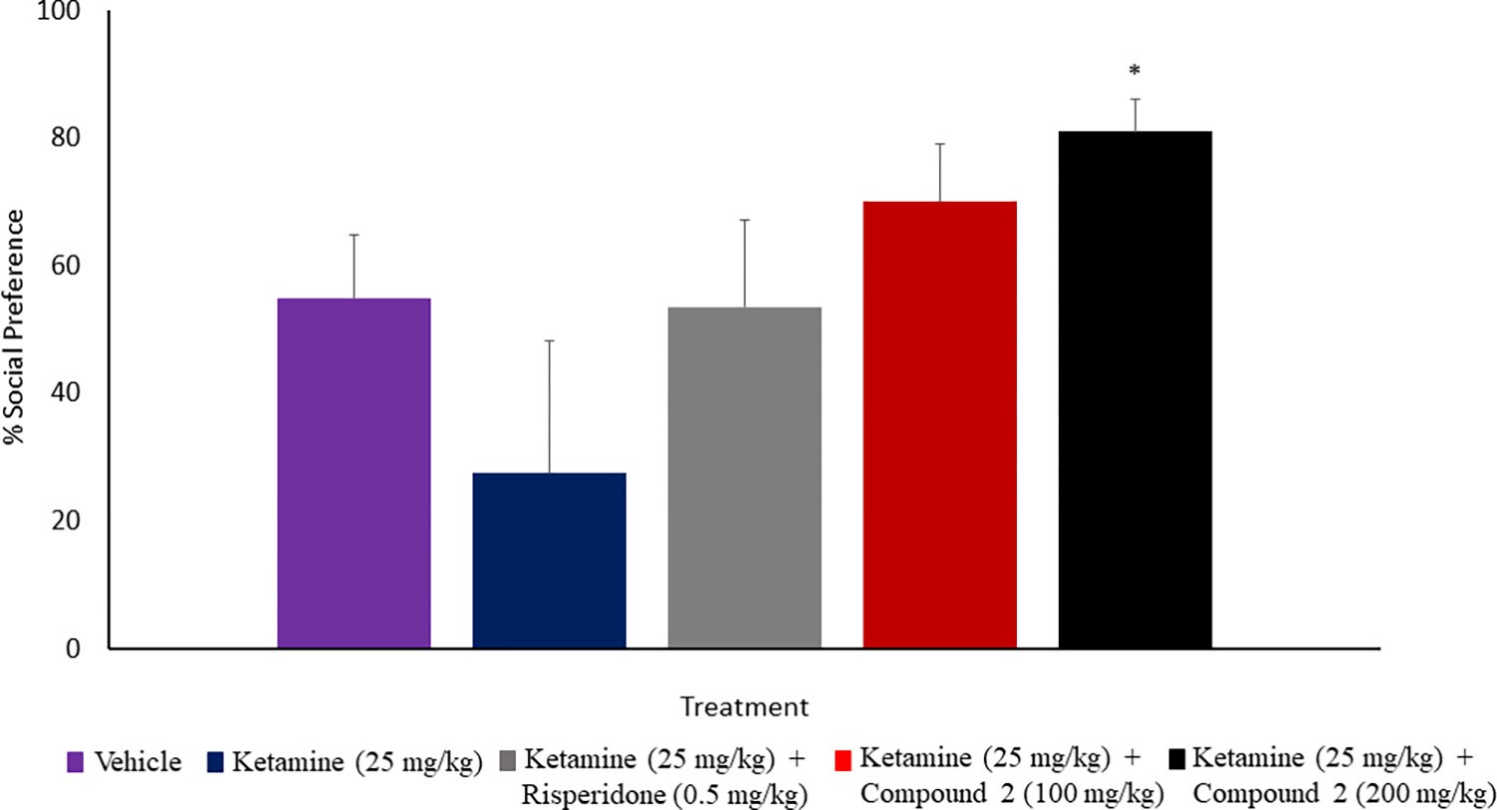
Effect of compound 2 on ketamine-induced social isolation. Rats were injected with ketamine for 10 days, then left undisturbed for 3 days. On day 14th, vehicle, risperidone or compound 2 was administered, and rats were tested for social preferences. Rats treated with 200 mg/kg compound 2 showed a significant increase in % social preference than rats treated with ketamine. Data are expressed as a mean ± S.E.M (n = 8–12) and analysed by One-way ANOVA followed by Tukey’s post hoc test, *p < 0.05 compared to the schizophrenia control group.

In order to determine whether inhibition of PDE1B and PDE10A has activity in a preclinical model of negative symptoms, SIT was conducted. A planned comparison was used to determine whether time spent in the social chamber and the empty chamber was different within each treatment group. Chronic injection of ketamine (25 mg/kg) for 10 days decreased the preference for social interaction when compared with the control and treatment groups, which suggests declines in social activity. The administration of compound 2 (200 mg/kg) significantly (p < 0.05) increased the social preference relative to the ketamine (25 mg/kg) group (Fig 5). The test rats in the compound 2 (200 mg/kg) group spent more time interacting with the unfamiliarised rat’s cage compared to the empty cage, which is in accordance with the preference of these rats for social interaction. Moreover, treatment with compound 2 (100 mg/kg) and risperidone (0.5 mg/kg) have also increased the preference for social interaction along with the control group rats performing at a comparable level. However, the increase was not significant (p ˃ 0.05) when compared with the ketamine (25 mg/kg) group. Although compound 2 (200 mg/kg) elicited a stronger social preference than compound 2 (100 mg/kg) and risperidone (0.5 mg/kg), the two groups have shown a strong trend toward the significant effect.

### PDE1B/PDE10A inhibitor rescued recognition memory deficit in ketamine-treated rats

**Fig 6:**
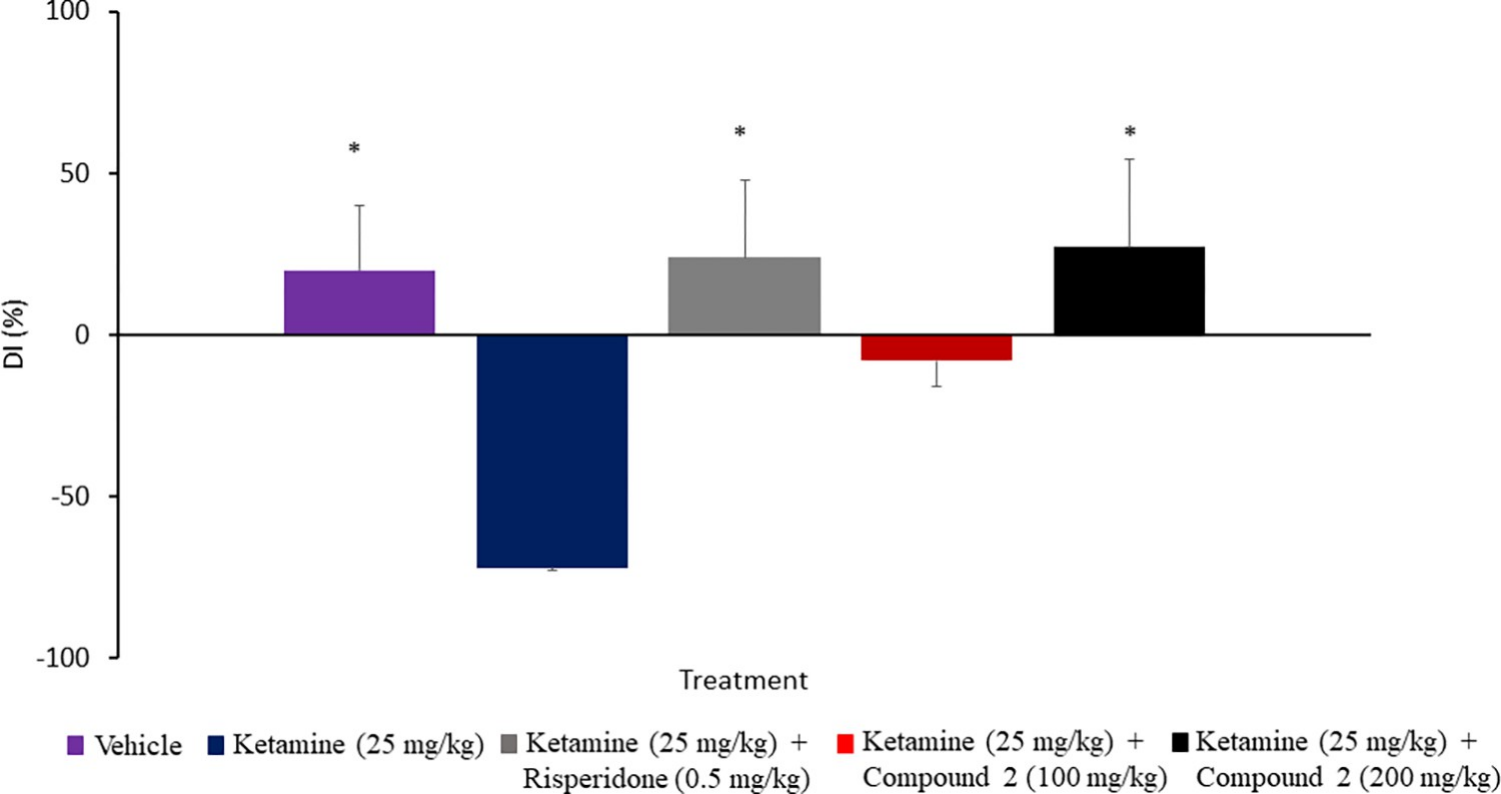
Effect of compound 2 on ketamine-induced non-spatial working memory impairment. Rats were injected with ketamine for 10 days, then left undisturbed for 3 days. On day 14th, vehicle, risperidone or compound 2 was given, and the rats were trained for 15 min to induce the recognition memory in the sample trial. After 24 h, rats were tested for memory retention in the choice trial. Rats treated with 200 mg/kg compound 2 showed significantly stronger memory retention than rats treated with ketamine and trained for 15 min. Data are expressed as a mean ± S.E.M (n = 8–12) and analysed by One-way ANOVA followed by Tukey’s post hoc test, *p < 0.05 compared to the schizophrenia control group.

Treatment with ketamine (25 mg/kg) significantly (p<0.05) decreased the recognition memory of the novel object, as indexed by the decrease in the discrimination index compared to the control group (Fig 6). Treatment with compound 2 (200 mg/kg) resulted in greater time exploring the novel object over the familiar object, suggesting it reverses the memory loss. Compound 2-treated rats (200 mg/kg) showed a significant increase (p<0.05) in the discrimination index relative to ketamine-treated rats. Treatment with compound 2 at a dose of 100 mg/kg did not result in a significant difference in the discrimination index; rats in this group spent almost similar time exploring both the familiar and novel objects. In contrast, ketamine-treated rats spent greater time exploring the familiar object over the novel object, demonstrating reduced retention of the previous learning experience due to impairment in the non-spatial recognition memory. Meanwhile, treatment with risperidone (0.5 mg/kg) and compound 2 (200 mg/kg) did not show any significant difference in the discrimination index when compared to the control group.

## Discussion

We have reported previously the identification of a dual inhibitor of PDE1B and PDE10A, compound 2, that has shown a potent inhibitory activity *in vitro* assay using recombinant human enzymes [25]. Here in the current study, we have presented data validating the safety and antipsychotic-like activity of compound 2. A fixed-dose procedure was employed to evaluate the safety profile of compound 2, as the compound is novel and no earlier studies on toxicity assessment were reported. The acute oral toxicity study revealed that there was no mortality, changes in wellness parameters (respiratory distress, excessive salivation, tremors and diarrhoea) and behaviour patterns, and no noticeable signs of toxicity observed in rats at 1 g/kg during the 14 days. The physical appearance of fur and eyes was found to be normal. Exposure to toxic substances is directly related to changes in body weight, which proves to be an indicative marker of toxicity. Body weight had increased in both control and compound 2 treated rats. All rats exhibited daily body increments without significant difference (p ˃ 0.05), indicating that compound 2 has no adverse effect on animals’ growth. Moreover, the rats were active throughout the study period corresponding to normal food and water consumption, which aided the weight gain.

Another index of the physiological and pathological status in humans and animals is the organ weights. The relative organ weight index is essential to find whether the organ has been exposed to a toxic substance or not. The vital organs, i.e. heart, liver, kidneys, spleen, and lungs, are the primary organs affected by metabolic reactions caused by poisonous substances [30]. Therefore, relative organ weight serves to diagnose the organ’s injury due to exposure to toxic materials. In this study, the relative organ weight of the vital organs showed a similar trend in both control and compound 2-treated groups. The administration of compound 2 did not affect the weight of the vital organs. Further, gross necropsy examination of the internal organs showed no detectable abnormalities, implying that compound 2 was non-toxic to the organs.

Analysis of serum biochemical parameters was also conducted to assess the effect of compound 2 on hepatic and renal functions in rats. The analysis of kidney and liver functions is indices of substance toxicity. The results have shown that compound 2 is safe and non-toxic to kidneys as there were no significant changes in the levels of the vital markers of renal dysfunction (urea and creatinine). The liver is also known as the metabolic hub of the body as it metabolites the potentially toxic substances that have been ingested. In the event of hepatic injury, the normal metabolic function of the liver will deteriorate, which results in the upregulation of the liver enzymes like ALT, ALP, and AST. ALT is related to liver function, while AST is found mainly in the heart, brain, skeletal muscles and kidneys. The significant elevation in the serum levels of these enzymes indicates liver damage [37]. In this study, the serum level of ALT, ALP and AST did not significantly differ (p ˃ 0.05) between control and compound 2-treated groups. Furthermore, as the liver is the main site of protein synthesis, the reduction in total protein and albumin may be indicative of hepatocyte damage. Our results showed that compound 2 did not affect the serum level of total protein or albumin in rats. Moreover, no significant differences (p ˃ 0.05) were observed between the control and compound 2-treated rats in terms of electrolytes levels such as sodium, potassium and chloride. The stability of electrolytes is essential for the normal function of cells and organs. According to OECD guidelines 423 (Annex 2), the results of the acute toxicity study classified compound 2 as category 4 [27]. Therefore, it can be concluded that compound 2 is tolerated up to 1 g/kg when administered at a single oral dose.

The potential pharmacological effect of compound 2 was evaluated through inhibition of ketamine-induced hyperlocomotion, social isolation and memory impairment in rats that are commonly used as predictive tests for antipsychotic-like activity [38]. Compound 2 was compared with risperidone, FDA-approved and available atypical antipsychotic, which is known to attenuate the hyperlocomotion caused by NMDA receptor antagonists in rodents. Ketamine-induced hyperlocomotion is commonly used as a rodent model for acute psychosis that assists in the prediction of the potential antipsychotic-like effect of test compounds in preclinical studies [24]. In this study, ketamine significantly increased the locomotor activity for rats compared to the control group rats, indicating hyperlocomotion. This finding was in agreement with the previous studies, which showed that ketamine administration results in an increase in locomotor activity in rats in the open-field test via an elevation in dopamine level in the striatum and cortex and inhibition of dopamine uptake [32, 33, 39]. Therefore, the ability of compound 2 to significantly inhibit the ketamine effects on the locomotion of rats, as evidenced by decreased total distance travelled, indicating the beneficial effects of compound 2 in the psychosis treatment probably by affecting the dopaminergic receptors in the indirect pathway; suggesting an antipsychotic-like property since the results were comparable to risperidone. Further, the administration of compound 2 only was devoid of sedative effect, based on the total distance travelled, which may provide an evidence of the advantage of compound 2 as a potential antipsychotic compound over the currently available antipsychotics. The marked sedative effect has been reported with the administration of typical and atypical antipsychotics, which is associated with the possible tendencies of extrapyramidal side effects. Extrapyramidal side effects result from the decreased dopaminergic input to the striatum [40]. Therefore, the preferential action of the new compounds against spontaneous motor activity may provide a predictive indicator of small or no tendency to induce extrapyramidal symptoms [41]. The administration of compound 2 alone did not decrease the total distance travelled in the open field arena. These findings provide strong evidence that the antipsychotic-like effects of compound 2 were not secondary to a motor impairment or behavioural suppression; rather, it might reflect the PDE10A inhibition in the indirect pathway and subsequent increases in the levels of striatal cAMP and cGMP. However, extrapyramidal symptoms should be studied further using a well-known test like the catalepsy test to evaluate the effect of compound 2 on motoric activity and coordination.

The negative symptoms and cognitive impairments are schizophrenia domains that are poorly treated by the available antipsychotics but are crucial for functional outcomes of the disease. PDE1B and PDE10A are expressed in the cortex and striatum, brain regions related to memory and cognition [15, 42]. Therefore, we have tried to understand the potential of this mechanism in negative symptoms and cognitive impairment. Compound 2 was capable of reversing the behavioural alterations induced by the repeated administration of ketamine in the rat model of schizophrenia that resemble negative and cognitive symptoms of this mental disorder in humans. Compound 2 at a dose of 200 mg/kg but not 100 mg/kg significantly attenuated ketamine-induced alteration in social interaction. The amount of time spent by compound 2-treated rats interacting with the unfamiliar rats was significantly higher compared to ketamine-treated rats. The neurochemical pathways involved in ketamine-induced deficit in social behaviour were related to the decrease dopamine transmission from the ventral tegmental area (VTA) to the prefrontal cortex, an area of the brain where hypodopaminergic functioning has been linked to negative symptoms and mood deficits [43–45]. Dopamine D_1_-receptors and NMDA-receptors cooperate with each other, in which the intensification of dopamine D_2_-receptor antagonists by dopamine D_1_-receptor agonists results in better NMDA transmission [46]. Furthermore, ketamine has interacted with the binding site of the phencyclidine (PCP) within the NMDA receptor channel complex and consequently has simulated all the behavioural phenotypes that resemble the PCP animal model of schizophrenia [47]. This finding supports the use of ketamine as an alternative tool to screen the tested agents with potential antipsychotic-like activity, especially against negative symptoms. However, the ability of compound 2 to reverse ketamine-induced social withdrawal might be mediated by its action as a PDE1B inhibitor in the prefrontal cortex and activation of dopamine D_1_-receptors, which in turn could mediate improvements in the negative symptoms and enhance social behaviour.

Moreover, the potential effect of compound 2 on cognitive impairments was evaluated by studying its effect on recognition memory that is known to be disrupted in schizophrenia in a novel object recognition test, a benchmark test of recognition memory in rodents. In this model, rodents explore two identical objects in the sample trial. After 24 h, the choice trial was conducted, in which rodents were presented with one familiar object and one novel object. The natural curiosity of the rodent pushed it to spend longer time exploring the novel object over the familiar object. Measuring the time spent by the animal exploring the novel object rather than the familiar object allows quantifying the memory of the animal. A compound that improves retention memory should augment animal’ performance by spending more time exploring the novel object over the familiar object [23]. Compound 2, vehicle or risperidone was administered 60 min prior to the sample trial, and rats were trained for 15 min to induce a weak memory for the familiar objects. Rats received 200 mg/kg compound 2 showed significantly improved recognition memory compared to ketamine-treated rats in a choice trial. The memory in 200 mg/kg compound 2-treated rats was comparable in magnitude to that observed in 0.5 mg/kg risperidone-treated rats and control group rats, in which the three groups spent significantly more time exploring the novel object as indicated by their DI. The efficacy of compound 2 in improving memory performance is likely to reflect the substrate selectivity of the PDE1 and PDE10A enzyme families. According to previous reports, cAMP and cGMP serve predominant roles in different phases of memory formation. The cAMP-specific PDE enzyme inhibitors, such as PDE4 inhibitors, are effective at promoting late consolidation [48, 49]. While the cGMP-specific PDE enzyme inhibitors such as PDE5 inhibitors are effective at enhancing early consolidation processes. Further, Inhibitors of PDE enzymes that regulate both cAMP and cGMP exert effects on both acquisition and consolidation of memories [48, 50]. In line with these reports, the potent effect of compound 2 on memory performance might be attributed to its dual inhibitory effect of PDE1B and PDE10A, which in turn regulate cAMP and cGMP and enhance both memory acquisition and consolidation. Further, PDE1B and PDE10A are highly expressed in brain neurons involved in memory and learning. PDE1B is abundantly expressed in the hippocampus, frontal cortex and striatum, whereas PDE10A expression is restricted to the striatum [49, 50]. The frontal cortex and striatum are interconnected through neuronal circuitry, where the striatum plays a general role in learning and cognition [51]. The overexpression of D_2_-receptors in the striatum altered the dopamine metabolism and D_1_-receptor signalling in the prefrontal cortex via VTA or thalamus, which results in deficits in prefrontal cortex-dependent cognition tasks. Therefore, increasing the D_1_-receptor signalling in the prefrontal cortex and striatum by PDE1B inhibitor and the striatal outputs to the cortex by PDE10A inhibitor may modulate the cortical activity through corticostriatal circuits and contribute to cognitive functions [52].

## Conclusion

Negative symptoms and cognitive deficits are the main complications with schizophrenia that substantially contribute to the diminished quality of life of patients with the disease, even when hallucination and delusions are controlled. The limitations of the currently available antipsychotics, in particular, lack of relief from the negative and cognitive symptoms, make the development of more effective therapeutic agents an unmet medical need. Herein, we have shown that a dual inhibitor of PDE1B and PDE10A, compound 2, prevented and reversed schizophrenia-like behavioural alterations induced by ketamine, suggesting the potential of compound 2 as a promising therapy for schizophrenia.

## Supporting information

S1 Table Acute oral toxicity record sheet for the vehicle and compound 2 treated rats. (DOCX)

S2 Table Serum biochemistry parameters of the vehicle and compound 2 treated rats in acute oral toxicity test. (DOCX)
