# Supplementary material for: Evaluation of the acute oral toxicity and antipsychotic activity of a dual inhibitor of PDE1B and PDE10A in rat model of schizophrenia
Source: PLoS One. 2022 Dec 1;17(12):e0278216. doi: 10.1371/journal.pone.0278216 (PMC9714703; doi:10.1371/journal.pone.0278216)
Supplement: S1 Table — (DOCX) [file pone.0278216.s001.docx]

**S1 Table. Acute oral toxicity record sheet for the vehicle and compound 2 treated rats.**

| **Parameters of behavioural study** | **Vehicle** | **Compound 2 (1 g/kg)** |
| --- | --- | --- |
| **Fur and skin** | 0/3 | 0/3 |
| **Eyes change** | 0/3 | 0/3 |
| **Salivation** | 0/3 | 0/3 |
| **Respiratory distress** | 0/3 | 0/3 |
| **Hypo/Hyperactivity** | 0/3 | 0/3 |
| **Tremors** | 0/3 | 0/3 |
| **Convulsions** | 0/3 | 0/3 |
| **Ataxia** | 0/3 | 0/3 |
| **Analgesia** | 0/3 | 0/3 |
| **Diarrhoea** | 0/3 | 0/3 |
| **Mortality** | 0/3 | 0/3 |

*Data presented as sign of toxicity/number of animals.
