# Supplementary material for: Evaluation of the acute oral toxicity and antipsychotic activity of a dual inhibitor of PDE1B and PDE10A in rat model of schizophrenia
Source: PLoS One. 2022 Dec 1;17(12):e0278216. doi: 10.1371/journal.pone.0278216 (PMC9714703; doi:10.1371/journal.pone.0278216)
Supplement: S2 Table — (DOCX) [file pone.0278216.s002.docx]

S2 Table. Serum biochemistry parameters of the vehicle and compound 2 treated rats in acute oral toxicity test

| **Biochemical parameter** | **Vehicle** | **Compound 2**  **(1 g/kg)** | ***P* value** |
| --- | --- | --- | --- |
| **Urea mmol/L** | 1.4 ± 0.0 | 1.3 ± 0.11 | 0.435 |
| **Creatinine μmol/L** | 33.66 ± 0.88 | 33.33 ± 0.33 | 0.742 |
| **Uric Acid μmol/L** | 230.33 ± 9.59 | 231.33 ± 33.76 | 0.979 |
| **Total protein g/L** | 76.23 ± 0.38 | 75.76 ± 1.21 | 0.734 |
| **Albumin g/L** | 45.60 ± 1.13 | 40.03 ± 2.99 | 0.157 |
| **ALP (alkaline phosphatase) IU/L** | 184.33 ± 42.83 | 295.00 ± 29.28 | 0.100 |
| **ALT (alanine aminotransferase) IU/L** | 4.13 ± 0.92 | 4.26 ± 1.63 | 0.947 |
| **AST (aspartate aminotransferase) IU/L** | 1.1 ± 0.100 | 1.03 ± 0.57 | 0.915 |
| **Sodium mmol/L** | 138.33 ± 0.66 | 138.66 ± 1.66 | 0.862 |
| **Potassium mmol/L** | 8.84 ± 0.26 | 8.21 ± 0.30 | 0.195 |
| **Chloride mmol/L** | 91.733 ± 1.51 | 91.00 ± 1.10 | 0.716 |

*Data are expressed as a mean ± S.E.M (n = 3) and analysed by independent samples t-test, p ˃ 0.05 for compound 2-treated group compared with the vehicle group.
